# Family Physicians’ Standpoint and Mental Health Assessment in the Light of COVID-19 Pandemic—A Nationwide Survey Study

**DOI:** 10.3390/ijerph18042093

**Published:** 2021-02-21

**Authors:** Tina Vilovic, Josko Bozic, Marino Vilovic, Doris Rusic, Sanja Zuzic Furlan, Marko Rada, Marion Tomicic

**Affiliations:** 1 Department of Family Medicine, University of Split School of Medicine, 21000 Split, Croatia; tvilovic@mefst.hr (T.V.); sanja.zuzic@dz-sdz.hr (S.Z.F.); ravnatelj@dz-sdz.hr (M.R.); 2 Department of Family Medicine, Split-Dalmatia Health Center, 21000 Split, Croatia; 3 Department of Pathophysiology, University of Split School of Medicine, 21000 Split, Croatia; josko.bozic@mefst.hr (J.B.); marino.vilovic@mefst.hr (M.V.); 4 Department of Pharmacy, University of Split School of Medicine, 21000 Split, Croatia; doris.rusic@mefst.hr

**Keywords:** COVID-19, knowledge, attitudes, practices, non-communicable disease, mental health, family physician, primary care

## Abstract

During the coronavirus disease 2019 (COVID-19) outbreak, family physicians (FPs) are the backbone of the healthcare system with considerable impact on the general population, and their well-being is of great importance. The aim of this investigation was to assess FPs mental health, as well as knowledge, attitudes and practices (KAPs) regarding the pandemic, and opinions on non-communicable disease (NCD) health care provided to patients. A cross-sectional study was carried out with a sample of 613 FPs. Anxiety and depression levels were estimated with the Hospital Anxiety and Depression Scale, subjective perceived stress with the Perceived Stress Scale, while trauma-related symptoms were assessed using the Impact on Event Scale-COVID19. KAPs toward the pandemic and opinions regarding NCD patients were evaluated with questionnaires accordingly. Results have shown that age (β = −0.02, *p* = 0.013) and personal risk of COVID‑19 (β = 1.05, *p* < 0.001) were significant independent correlates of the knowledge score. A total of 87.7% FPs expressed moderate/high perceived stress, 45.2% moderate/severe trauma-related symptoms, 60.4% borderline/abnormal anxiety levels, and 52.4% borderline/abnormal depression levels. Knowledge score was an independent predictor of perceived stress (β = −0.33, *p* = 0.023) and anxiety (β = −0.31, *p* = 0.006) levels. Limited accessibility to healthcare services and decreased number of newly-diagnosed NCD cases were mostly agreed on. The pandemic puts a considerable strain on FPs mental health, as well as on public health measures, due to the decreased overall quality of NCD patient health care. Educational programs may bridge the gaps between FPs’ knowledge. Thus lowering anxiety and improving patient care.

## 1. Introduction

In late December of 2019, the Chinese city Wuhan recorded the first case of infection with the novel single-stranded RNA coronavirus—severe acute respiratory syndrome coronavirus 2 (SARS-CoV-2), which causes coronavirus disease 2019 (COVID-19) [1]. This was followed by its rapid global spread, and on 30 January 2020, the World Health Organization (WHO) officially declared it a Public Health Emergency of International Concern, while on 11 March 2020, it was established as a pandemic disease [2]. Regarding the Republic of Croatia, to date (20 January 2021), over 225,000 cases and 4700 confirmed deaths have been recorded [3,4]. Currently, Croatia has about 1140 total deaths per 1 million population, with a rapid rise during October and November 2020, which puts it alongside states in Europe that could be considered most affected by COVID-19 [3,4].

Since the beginning of the epidemic, in many countries strict regulatory government measures were introduced in order to stop further transmission of the virus, while providing adequate care for the infected patients [5,6]. Some of the measures that were effective in Croatia during the current study data acquisition were a ban on all public gatherings with more than 25 people, a limitation of private gatherings to 10 people, a ban on all wedding celebrations, limited public transportation capacity, and cessation of work om various restaurants, coffee shops, fitness centers and sport facilities. Moreover, during a brief period in late December, traveling between Croatian counties was banned for everyone without specialized permits. Life was re-organized thoroughly, and shifted to a “new normal” paradigm, with major changes in social interaction and overall daily living, but consequently and increased sense of fear and other mental health disorders [7,8].

In order to cope with all the changes this pandemic has brought, a complete reorganization of the healthcare system took place, and shifted its focus to management and triage of COVID-19 patients [9,10]. Specialized COVID-19 centers rapidly emerged throughout the country, and one of the main carriers of the logistical and adaptive burden that emerged were family physicians (FPs). On the frontline of the pandemic, and point of first contact with the healthcare system, FPs represent a crucial factor in providing a common link between all other healthcare services [10,11,12,13,14]. However, at the same time, they are also managing most of the COVID-19 patients, all of which is causing profound changes in primary care functioning [10,14].

In these challenging times, with increased skepticism and a large amount of fake and misinterpreted information, it is of utmost importance to properly educate the population regarding COVID-19’s characteristics and prevention. As FPs have numerous and comprehensive roles in society [15,16], they are often regarded as educators, with a special relationship with their patients, who see them as someone whom they can trust [12,17,18]. Hence, their overall knowledge about and behavior toward the pandemic is crucial in order to properly convey evidence based information to their patients. However, there is very limited information regarding these features published in the literature, to the best of our knowledge.

Furthermore, as studies have shown that FPs have an increased risk of high stress and burnout even before the pandemic [19,20], additional COVID-19 related pressure could make a further deleterious impact on FPs’ mental health. Although the negative impact of the pandemic on healthcare workers’ mental health is already well established in numerous studies, there is still scarce information involving FPs [21,22]. Relevant recent work that explored this specific topic includes a study by Amerio et al., which reported negative mental health outcomes in a sample of Italian primary care physicians [23], and a study by Monterrosa-Castro et al., which reported symptoms of generalized anxiety disorder in a Colombian sample [24].

Moreover, with all the COVID-19 related changes in the healthcare system, and even more workload put on FPs, it is possible that the quality and continuity of primary healthcare is severely obstructed for other patients with non-communicable diseases (NCDs) in need of chronic care [25,26]. However, attitudes from the FP perspective on this aspect of the pandemic have not been studied yet, to the best of our knowledge.

Therefore, the main goal of the current study was to investigate knowledge, attitudes and practices (KAPs) of FPs toward the COVID-19 pandemic with assessment of trauma-related symptoms, perceived stress, anxiety and depression levels. Further connections between mental health characteristics and COVID-19 knowledge, gender and perceived personal risk from COVID-19 were explored. An additional goal was to investigate FPs’ attitudes concerning the impact that the COVID-19 pandemic has had on NCD patients.

## 2. Materials and Methods

### 2.1. Study Design and Participants

In this cross-sectional survey study, all working family physicians in the territory of the Republic of Croatia, with a minimum of two years of practice experience, were deemed eligible for participation. In this way, we excluded FPs that would probably not have enough practice experience before the pandemic started to have a proper experience-based estimation of attitudes regarding health care provided to NCD patients. Data was collected using a comprehensive survey that was shared via Google Forms^®^ online application, in a setting which guaranteed anonymity of provided answers. The survey was shared during the “second wave” of the COVID-19 pandemic, from 30 November 2020 to 15 January 2021. A total of 626 FPs submitted responses to the survey, however, 13 were excluded due to stated working experience of under two years. All relevant information regarding the study was posted in the accompanying mail and introduction section of the survey, and potential questions could be asked via e-mail. Submitted response was considered as informed consent, as this was also highlighted to the participants. Links to the survey were distributed via e-mail to family medicine practices in the country, and it was sent to members of all relevant Croatian family medicine associations. Overall response rate was 26.4%.

Participation in the study was completely voluntary, without any kind of compensation for the participants. Furthermore, the survey did not gather any data that could be used to identify physicians. Research was performed according to the newest version of the Helsinki Declaration, and was approved by the Ethics Committee of the University of Split School of Medicine and the Ethics Committee of the Health Centre of the Split-Dalmatia County.

For the purposes of this study, a free online Surveymonkey^®^ sample size calculator was used. According to the official data of the Croatian Institute for Health Insurance, our population of interest consisted of a total of 2324 registered FPs in the Republic of Croatia. Using the calculator, a minimum sample needed for this study was 330 FPs, according to 95% confidence interval and 5% margin of error. To ensure additional power, we collected a substantially larger sample of FPs.

### 2.2. Survey

For the purposes of this investigation, a comprehensive questionnaire was used for data gathering, that consisted of three main sections. The first section included 10 items that explored basic demographic data of the FPs, including questions about personal experience with COVID-19. The items gathered information on subjects’ age, gender, work experience, number of patients in care, occupation (family physician vs. family medicine resident) and practice localization (urban vs. rural/island area). Data on number of patients in practice was collected from a total of 567 FPs (as some were not yet assigned officially with their own practice) as the carriers of the primary care team. Furthermore, the rest of the first section included questions regarding self-assessment of the increased personal risk for COVID-19 (age over 60 or presence of relevant NCDs), work experience in a COVID-19 center, main source of COVID-19 related information, and self-assessment of confidence regarding personal COVID-19 knowledge.

The second section of the survey explored FPs’ mental health by assessing levels of anxiety, depression, perceived stress and trauma-related symptoms, while the third survey section investigated FPs’ knowledge, attitudes and practices regarding the COVID-19 pandemic, and opinions regarding the impact that the pandemic has on NCD patients that require chronic care.

Prior to the start of the data collection, the survey was pilot-tested on 15 family physicians and eight family medicine residents to assess the intelligibility and readability of all included sections. None of the physicians reported any difficulties in answering included items, and they found each to be appropriate to the current situation, with average time for survey submission of 15–20 min. The final survey version was then forwarded to the main FP population.

### 2.3. Mental Health Assessment

The Hospital Anxiety and Depression Scale (HADS), developed by Zigmond and Snaith, was used for the assessment of anxiety and depression levels in our population [27]. This is one of the most commonly used tools for this purpose, already used on Croatian population samples, with good internal consistency scores [28,29]. It is a 14-item scale, divided into Anxiety (HADS-A) and Depression (HADS-D) subscales, each comprising seven items that explore how the subjects have been feeling during the past week. Possible responses for each of the statements were given in the form of a 4-point Likert scale, that were assigned from 0 to 3 points. Therefore, the total HADS-A and HADS-D scores range from 0 to 21 points, with higher scores indicating more severe levels of anxiety or depression. Given the final result of the scales, a total score of 0 to 7 points was considered as “normal”, 8 to 10 was considered as a “borderline case”, while 11 to 21 was classified as an “abnormal case” of anxiety or depression. In our population sample, Cronbach’s alpha coefficient for the HADS-A subscale was 0.89, and for the HADS-D subscale 0.87, indicating good internal consistency.

The Impact on Event Scale with Modifications for COVID-19 (IES-COVID19) was used to determine levels of trauma-related stress symptoms [30]. This is a self-reported scale based on the Impact on Event Scale (IES)—a well-established, validated tool for the assessment of subjective distress reactions after various traumatic events [30]. It correlates well with Post Traumatic Stress Disorder (PTSD) symptoms, though it should be used with caution, and not as a definitive tool for PTSD diagnosis [30,31]. Vanaken et al. made specific modifications to the original IES scale in order to fully refer to the current COVID-19 outbreak, showing good measures of test-retest reliability and internal consistency [30]. The scale comprises 15 items, with seven items measuring the intrusive dimension, and eight items measuring the avoidance dimension of trauma-related psychological responses. Questionnaire responses are categorized via a 4-point Likert scale in which respondents rated the frequency with which items had occurred during the last week (0 not at all, 1-seldom, 3-sometimes, 5-often). The total sum of the responses determined the overall score, ranging from 0 to 75, with higher scores indicating more severe distress levels related to the COVID-19 pandemic. As suggested for the IES scale, according to the total score, respondents were categorized into four different ranges: “subclinical” (0–8), “mild” (9–25), “moderate” (26–43) and “severe” (≥44), with a score of 26 being the cut-off point over which significant clinical reaction is documented [30,31,32]. To ensure the quality of the IES‑COVID19 translation, a back translation technique was used by an English language professional. In our sample, the IES-COVID19 scale has shown excellent reliability, with a Cronbach’s alpha coefficient of 0.92.

The Perceived Stress Scale (PSS) is a well-validated, commonly used psychological instrument for measuring subjectively perceived stress levels [33]. It can be interpreted as a measurement of which external demands of life seem to exceed the respondent’s ability to cope, as well as of the perception of life’s overloaded, unpredictable, and uncontrollable features [33,34]. It is a 10 item scale that explores the frequency of specific feelings and thoughts during the past month, and it can be administered in any subpopulation group and situation since all of the items are structured in a general way [33,34]. A Likert scale is used to assess the answers, and this ranges from 0 (never) to 4 (very often), with a maximal possible score of 40 points. For this study, a score ranging from 0 to 13 was considered as low perceived stress, a score between 14 and 26 as moderately perceived stress, while a score from 27 to 40 was considered as high perceived stress. PSS has already been adapted and validated in the Croatian population [35,36], while it showed excellent internal consistency in our sample too, with Cronbach’s alpha coefficient of 0.91.

### 2.4. Knowledge, Attitudes and Practices Assessment

Our population’ KAPs toward the COVID-19 pandemic, as well as opinions on the pandemic’s impact on NCD patients were assessed with a questionnaire that was developed at The Department of Family Medicine, University of Split School of Medicine, by two family physicians and two family medicine residents after an extensive and detailed review of all available literature. For this purpose, knowledge was assessed with a 20-item questionnaire, while attitudes and practices toward COVID-19, as well as opinions regarding NCD patients, were each assessed with a 15-item scale.

The final version of the knowledge test consisted of 20 questions that addressed various, comprehensive COVID-19 features—COVID-19 symptoms and complications, transmission characteristics, therapy management, current epidemiological guidelines, general epidemiological features and comparison to seasonal influenza mortality. Questions were organized as statements with three possible answer options—“true”, “false” and “I don’t know”. For each question, 1 point was given if answered correctly, meaning the total score ranged from 0 to 20 points, with larger scores indicating better overall COVID‑19 knowledge. The test was partly adapted as a combination of several different studies that had explored the same topic [37,38,39,40], all of which showed acceptable coefficients of internal consistency. Afterwards, it was expanded and modified in order to address wider COVID-19 knowledge that could also be a point of interest to the general population, and to be more applicable to the current pandemic and top primary care. To ensure the quality of the included questions, further detailed research was conducted, with emphasis on official Croatian Institute of Public Health (CIPH) guidelines and the WHO questions and answers section, with official up-to-date information on COVID-19. The draft version of the modified knowledge test included 27 questions that were additionally evaluated by two experts in the fields of Public Health and Internal Medicine, each having frontline experience in managing the pandemic. Finally, a consensus was made on the exclusion of seven questions which had potentially ambiguous answers, or that were found to be substantially unrelated to COVID-19 knowledge in primary care. Internal consistency of knowledge in our sample was acceptable, with Cronbach’s alpha coefficient of 0.75.

The attitudes scale comprised a total of 15 items that had been mostly adapted and modified from the existing literature [37,41,42,43,44]. A draft version of the attitudes scale consisted of 14 items, from which seven items were disregarded due to the perceived low suitability for FPs. However, an additional eight items were introduced in order to explore attitudes regarding COVID-19 vaccination, education and prevention in more detail. Responses could be selected on a 5-point Likert scale, ranging from “fully disagree” to “fully agree”.

Frequencies of practices followed during the COVID-19 pandemic were assessed using a 15 item scale, adapted from the previous literature after detailed research [37,39,40,41,42,44]. Items explored preventive behaviors of FPs in a professional, as well as in a private setting, with two items collecting information regarding education of patients and friends and family. Respondents could choose an answer on a 4-point Likert scale that most closely described the frequency of the practice followed, ranging from “never” to “always”.

Finally, attitudes regarding the impact that the pandemic has on NCD patients were evaluated with a 15-item scale, with answers organized via a 5-point Likert scale (ranging from “fully disagree” to “fully agree”). Considering the extremely limited information in the available literature regarding such attitudes in primary care, as well as attitudes of healthcare workers in general, a newly developed questionnaire was introduced. Items were organized to cover wide a range of COVID-19 impact features, including availability of primary care and other medical services to NCD patients, characteristics of exacerbation and potential change in illness management, NCD patients’ following of the epidemiological guidelines, and impact on unhealthy habits. A draft version included a total of 21 items; however, six items were removed after further evaluation by two additional FPs. Consensus was made on excluded items which were found to have low intelligibility, or were found to be inappropriate to the current pandemic.

Proper translation of every item in the KAP questionnaires was established with a back translation technique conducted by an English language expert.

### 2.5. Statistical Analysis

Statistical analysis was performed with the MedCalc statistical package for Windows (version 19.1.2, MedCalc Software, Ostend, Belgium). Categorical variables were presented in the form of whole numbers (*N*) and percentages (%), with Chi-squared (χ^2^) test used for measuring statistical differences. Furthermore, normality of data distribution for continuous variables was assessed with the D’Agostino-Pearson test, for all variables not showing normal distribution. Therefore, variables were presented as median and interquartile range, with statistical differences between groups tested via Mann-Whitney U test. Moreover, correlations between mental health questionnaire scores and other continuous variables were tested with Spearman’s rank correlation coefficient. Finally, in order to determine significant factors independently associated with mental health and knowledge questionnaire scores, multiple linear regression analysis was used. For this purpose, enter algorithm was selected, with unstandardized beta coefficients (β), standard errors (SE), t‑values and *P*-values reported. All assumptions for the use of multiple linear regression were satisfied. In the current study, the level of statistical significance was set to a value of *p* < 0.05.

## 3. Results

### 3.1. Baseline Information and COVID-19 Knowledge Characteristics

Data were collected from a total of 613 FPs (491 females and 122 males), of which 13.7% were family medicine residents. Median age of the population was 44.0 (35.0–55.0), with work experience of 13.0 (7.0–26.0) years. The number of patients in care per practice was 1747.0 (1500.0–1983.0), according to the 567 FPs’ recorded answers. Personal risk of COVID-19 was perceived as higher in 28.1% participants, while 67% had experience working in a COVID-19 center, without significant differences according to gender (Table 1). Furthermore, 40% of FPs perceived themselves to be confident in their personal COVID-19-related knowledge, while the main source of information regarding COVID-19 was official WHO or Croatian Institute of Public Health (CIPH) guidelines (60%).

**Figure 1:**
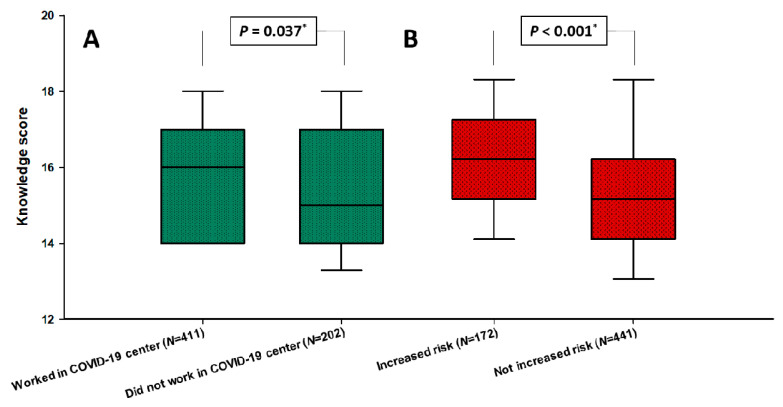
Differences in knowledge scores between family physicians according to experience working in COVID-19 center (**A**) and increased perceived personal risk of COVID‑19 (**B**). COVID-19—Coronavirus disease 2019. * Mann-Whitney U test.

Overall knowledge score of the entire population was 16.0 (14.0–17.0), without significant differences according to gender (Table 1). However, knowledge score was statistically higher in FPs with experience working in a COVID-19 center in comparison with FPs without such experience (16.0 (14.25–17.0) vs. 15.0 (14.0–17.0), *p* = 0.037), as well as in FPs with perceived increased risk of COVID-19 in comparison to those without perception of higher risk (16.0 (15.0–17.0) vs. 15.0 (14.0–16.0); *p* < 0.001) (Figure 1).

The study population had a ≥90% correct answers in a total of 10 questions, while the best answered question was about persistence of symptoms after acute phase of COVID-19 (K20, correct answer rate 99.2%). Lowest correct answer rate was to the question regarding increased risk of COVID-19 in pregnant women (K19, correct answer rate 15.8%), while important questions on disease symptoms (K3), treatment (K14) and comparison to seasonal influenza mortality (K17) had correct answers rates of 68.4%, 62.8 % and 59.1%, respectively. The detailed correct answer rate for all included questions in the knowledge test can be seen in Table S1.

Furthermore, in a multiple linear regression analysis model, age (β = –0.02, SE = 0.007, t = –2.48, *p* = 0.013) and increased personal risk of COVID-19 (β = 1.05, SE = 0.18, t = 5.63, *p* < 0.001) were found to be significant independent correlates of total knowledge score, when computed alongside other relevant socio‑demographic parameters (gender, practice localization, work experience in a COVID-19 center).

### 3.2. Mental Health Characteristics and Associations with Other Parameters

Mental health questionnaire analysis revealed that 87.7% FPs had increased perceived stress levels (63.6% moderate and 24.1% high), 45.2% exhibited clinically significant trauma related symptoms (27.7% moderate and 17.5% high), while 60.4% had increased levels of anxiety (19.6 borderline cases and 40.8 abnormal cases), and 52.4% of depression (30.2 borderline cases and 22.2 abnormal cases). Moreover, considerably greater mental health disturbance symptoms were found in female FPs in comparison to male FPs, with significantly higher total scores in all used questionnaires, as well as greater percentages in more severe questionnaire categories (*p* < 0.05). Detailed information regarding mental health characteristics according to gender can be found in Table 2.

**Figure 2:**
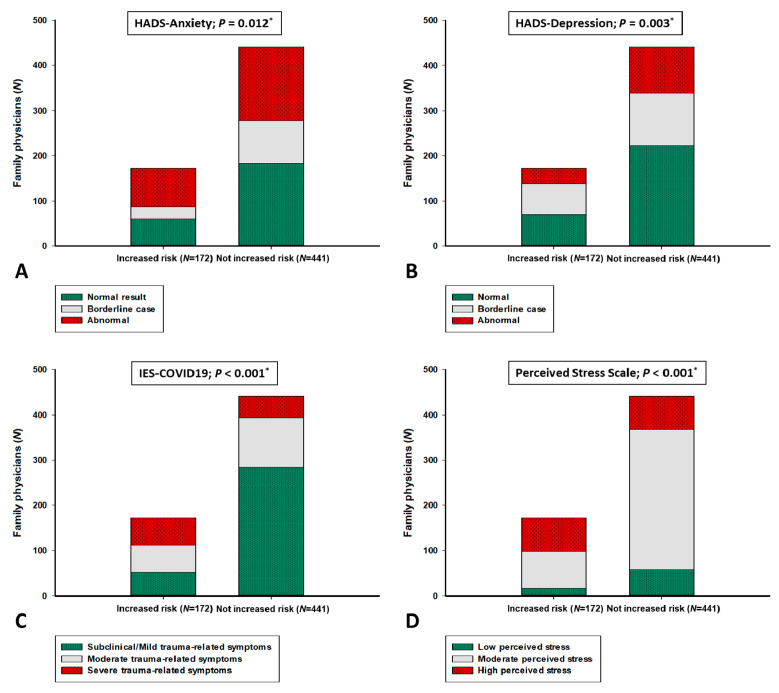
Mental health questionnaire categories according to increased personal risk of COVID-19. COVID-19—Coronavirus disease 2019; IES-COVID-19—Impact on Event Scale-coronavirus disease 2019; HADS—Hospital Anxiety and Depression Scale. * chi-square test. (**A**) HADS-Anxiety categories; (**B**) HADS-Depression categories; (**C**) IES-COVID19 categories; (**D**) Perceived Stress Scale categories.

Furthermore, FPs with increased personal risk of COVID-19 have shown significantly lower percentages in the mental health questionnaire categories without expressed symptoms when compared to FPs without the risk, according to HADS-A (34.9 vs. 41.5%), HADS-D (40.1 vs. 50.6%), IES COVID19 (30.2 vs. 64.4%) and PSS (9.9 vs. 13.2%) scales (Figure 2).

**Figure 3:**
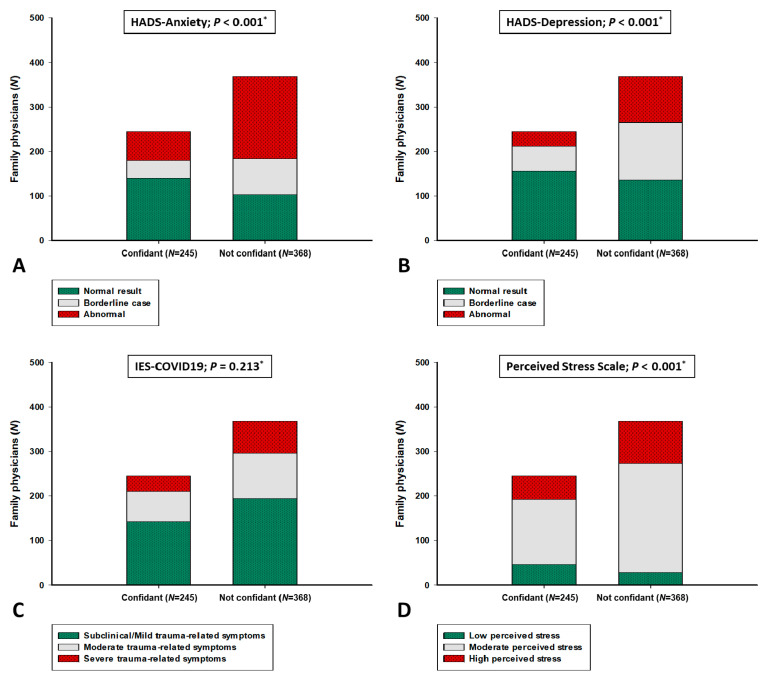
Mental health questionnaire categories according to self-assessment of personal confidence regarding overall COVID-19 knowledge. COVID-19—coronavirus disease 2019; IES-COVID-19—Impact on Event Scale-coronavirus disease 2019; HADS—Hospital Anxiety and Depression Scale. * chi-square test. (**A**) HADS-Anxiety categories; (**B**) HADS-Depression categories; (**C**) IES-COVID19 categories; (**D**) Perceived Stress Scale categories.

In our population sample, age positively correlated with PSS (r = 0.106, *p* = 0.009), HADS-D (r = 0.111, *p* = 0.006) and IES-COVID19 (r = 0.195, *p* < 0.001) scores. Analyses have also revealed that number of patients in practice positively correlated with PSS (r = 0.128, *p* = 0.002), HADS-A (r = 0.119, *p* = 0.003) and HADS-D (r = 0.135, *p* = 0.001) scores. Evaluating the association of mental health symptoms with objective knowledge, a significant negative correlation was found between total knowledge score and HADS-A score (r = −0.131, *p* = 0.001) (Table 3). Moreover, FPs that were found to be confidant in personal COVID-19 knowledge were significantly more represented in mental health questionnaire categories without expressed symptoms according to HADS-A (57.1 vs. 28.0%), HADS-D (63.7 vs. 37.0%) and PSS (18.8 vs. 7.9%) scales (Figure 3).

Finally, multiple linear regression models confirmed that increased risk for COVID‑19 and female gender were independent correlates of each of the used questionnaire scores, when computed with other socio-demographic factors and knowledge score. Furthermore, working experience in a COVID-19 center was an independent correlate of PSS (β = 1.77, SE = 0.57, t = 3.13, *p* = 0.002) and HADS-D (β = 1.24, SE = 0.37, t = 3.36, *p* < 0.001) scores, while knowledge score was shown to be an independent predictor of PSS (β = −0.33, SE = 0.15, t = −2.27, *p* = 0.023) and HADS-A (β = −0.31, SE = 0.11, t = −2.76, *p* = 0.006) scores. Detailed analysis of the regression models is shown in Table 4.

### 3.3. Attitudes and Practices

Evaluation of attitudes items revealed that the majority of our population was worried about the negative impacts of the pandemic. Therefore, 76.5% agreed on concerns about social isolation, 82.7% were concerned about the posed risk to family and friends, while 70.5% deemed personal protective equipment (PPE) was not available in sufficient amounts. However, FPs had positive attitudes regarding influenza vaccination (70.1%) and COVID-19 vaccination being responsible for significant epidemic reduction (77.5%), while 51.4% agreed on getting vaccinated in the next six months. Finally, a majority of participants agreed on items regarding education, as 83.4% regularly follow COVID-19 updates, 66.2% have read COVID-19-related scientific articles, while 74.7% would like to attend more educational seminars. Furthermore, 43.7% of the FPs deem that there is increased antibiotics prescription in comparison with time before pandemic. Attitude items and detailed responses are shown in Table S2.

Furthermore, participants have shown good following of preventive COVID-19 behaviors, as “often” and “always” were the most frequently chosen answers in items regarding hand hygiene, social distancing and patient management. Furthermore, 95.3% of FPs seem to educate friends and family regarding COVID-19 prevention, and 96.6% their patients. Table S3 includes detailed answers on all practice items.

Analysis of attitudes regarding the pandemic’s impact on NCD patients revealed that our participants agreed on items concerning limited accessibility to FPs (50.1%), specialist conciliatory examinations (80.7%) and diagnostic procedures (80.6%). Moreover, 72% of FPs deem that there is a decrease in newly diagnosed NCDs, 9.8% think pandemic aggravates the availability of chronic therapy, while 74.5% indicate that they have less time to devote to NCD patients. Furthermore, 26% of our subjects agrees that illness exacerbation was experienced during the pandemic, and 31.8% that chronic therapy is received less regularly in comparison with the time before the pandemic. Detailed attitude answers related to NCD patients’ care are included in Table S4.

## 4. Discussion

In this nationwide survey study, we addressed FPs’ KAPs toward the COVID-19 pandemic, as well as mental health characteristics and opinions regarding the impact that the pandemic has on patients with NCDs.

Results have shown that the majority of FPs in our population have prominent symptoms of some form of mental health disturbance. According to analyzed questionnaires, 87.7% have moderate or high levels of perceived stress, 60.4% are in the borderline or abnormal anxiety category, 52.4% in the borderline or abnormal depression category, while 45.2% of FPs present themselves as having experienced a significant psychological impact regarding the COVID-19 situation. Furthermore, multiple linear regression analysis has shown that significant independent predictors for all the overall scores were increased personal risk of COVID-19 and female gender. Moreover, previous experience working in a COVID-19 center was an independent predictor for PSS and HADS-D scores.

Disturbingly high rate of exhibited traumatic stress symptoms was observed, which is in accordance with theories that the COVID-19 pandemic could even be characterized as a mass traumatic event [45]. Amerio et al. have also investigated mental health characteristics in a sample of Italian FPs (*N* = 131) during the pandemic, in March and April 2020 [23]. A total of 22.9 % FPs reported moderate to severe depressive symptoms, and those participants had significantly higher severity of anxiety and insomnia symptoms in comparison to FPs with absent or mild depressive symptoms. Furthermore, according to Monterrosa‑Castro et al., around 40% of 531 Colombian FPs had symptoms of generalized anxiety disorder during late March 2020 [24]. Our population has shown a significantly greater rate of FPs that presented themselves with depressive and anxiety symptoms than in the aforementioned studies, and these differences could be present because our study was conducted after almost a year of constant psychological strain caused by the pandemic. Moreover, most of the data for the present study was collected during the peak “second wave” of COVID-19 in late November and December of 2020, where Croatia was one of the European countries with a highest 14-day case notification rate per 100,000 population and 14‑day death rate per 1 million population [46].

During these challenges in 2020, a number of different studies explored the deleterious impact of the current pandemic on healthcare workers’ mental health [21,22,47]. In a systematic review analysis that investigated 59 different studies, the main reported predictors of mental health problems were personal exposure to COVID-19 and female gender [21]. These results are in accordance with results from this study, where personal risk, work in a COVID-19 center and female gender were the main independent predictors of mental health questionnaire scores. However, it is important to emphasize that none of the aforementioned studies was conducted in a primary care setting. It is possible that work in a COVID-19 center could affect FPs’ well-being in the current situation due to the increased emotional burden and mental strain caused by constant managing of infected patients firsthand. Furthermore, since it is established that women have increased risk of anxiety, depression and PTSD when compared to men in general, and that the female gender is more sensitive to interpersonal relationships, it is possible that a newly-presented stressful situation like pandemic management would only emphasize these existing gender differences in FPs [48,49,50].

Studies that explored FPs’ mental health before the pandemic have shown that FPs already had increased risk of chronic stress, burnout and poor mental well‑being [19,20,51]. With all the additional changes in the healthcare system that FPs needed to adapt to, the increased workload, care for COVID-19 patients, and general fear that emerged during the last year, it is not surprising that mental health well-being could have deteriorated substantially.

Further analyses have shown that HADS-A and PSS scores had a significant negative association with total knowledge score, as confirmed by multiple linear regression. Moreover, results revealed that FPs with positive confidence in their knowledge were significantly less represented in abnormal HADS-A, HADS-D and PSS categories. Information regarding knowledge and mental health association is limited, with only a few studies partly exploring this topic in a general population sample during the COVID-19 pandemic [8,28]. However, just as in the current study, provided results indicate that knowledge about some aspects of COVID‑19 could potentially be regarded as a protective measure against negative mental health symptoms [8,29]. In the current scenario, it is possible that FPs with higher overall knowledge levels have studied the disease and guidelines more carefully and have more confidence in their practical abilities and disease management. Therefore, with higher levels of perceived security, they could be less affected by negative impacts on mental health. These results can be interpreted as a guideline for possible future epidemics or other newly emerged challenges. With FPs being a crucial link in the healthcare system, emphasis should be on their comprehensive education, not only for proper guidance of their patients, but to preserve their own mental health too.

In the current study, FPs have shown moderately good general knowledge regarding COVID‑19, with increased personal risk for COVID-19 and younger age being the independent correlates of total score levels. However, it is important to note that the pandemic had been active for nearly a year at this time, and some questions of importance that relate to educating and treating the general population could have been answered more correctly. Correct answer rate for the question concerning the fact that running/stuffed nose and sneezing are not common symptoms of COVID-19 was only around 68%, and for the question about antibiotics and standard antiviral drugs not being first therapy of choice for COVID-19, the correct answer rate was only around 63%. These results imply that a large proportion of FPs could be misguided in basic patient counselling, triage and management. Moreover, actions accordingly could induce an additional impact on irrational antibiotic use and increased antimicrobial resistance [52], especially concerning the fact that around 44% of our population deems that antibiotics are being prescribed in increased amounts during the pandemic in a primary care setting.

Furthermore, Gokdemir et al. investigated the knowledge levels of 240 FPs from eight different countries, and the results showed good knowledge regarding the transmission and symptoms of the virus, similar to that in our population [53]. Hussain et al. also showed, in a population of primary healthcare providers, good knowledge levels regarding various aspects of COVID-19, while 68.8% of participants disagreed with the use of antibiotics as a preventive measure. This result could be seen as according to our own, with a remark that the study was conducted in March and April 2020 in tertiary care hospitals, and not in primary care [42]. In a recent review by Puspitasari et al., analysis of seven different studies also showed good levels of knowledge among healthcare workers, with correct answer rates similar to our results [54]. However, it is difficult to compare these results directly, as every study used a different type of knowledge questionnaire.

In this study, around 75% of the FPs expressed a desire to attend more educational programs and seminars regarding COVID-19. In addition, the majority seem to regularly seek new information about COVID-19, and have read scientific articles about this topic. A study by Nejasmic et al. showed that Croatian FPs express strong positive attitudes toward the promotion and usage of evidence based medicine, while the majority (80%) reported a lack of time in searching for relevant information [55]. As this study was conducted before the pandemic, it is evident that in the present scenario, with significantly increased workload, FPs have even less time to look for proper information. Moreover, they have positive attitudes toward vaccination for seasonal flu (around 70%), and to a lesser extent for COVID-19 (around 51%). According to this information, we can conclude that more educational programs and seminars should be organized for FPs, as they have a willingness and need to educate themselves further regarding these crucial topics, but supposedly lack sufficient opportunities. These programs should also have been incorporated from the early phases of the pandemic, as knowledge and attitudes of FPs could have an important impact on the behavior of the population in general, but especially regarding key factors, such as vaccination [12,17,18].

Our population expressed significant concern due to possible contagion, social isolation, transmitting the risk to their families and lack of the PPE. Similar concerns were recorded in a population of other European FPs [53], dental practitioners [56] and hospital health-care workers [37,44]. These results show that there is a similar level of concern and fear in this healthcare population due to COVID-19, with a probable negative impact on overall mental health, as is shown in the current study. Being on the frontline of the pandemic, these concerns could be an additional mental burden on already worn-out FPs. Additionally, delivery of sufficient amounts of PPE to FPs should be one of the top priorities during this pandemic, as in possible future epidemics, as perceived insufficiency could be connected with common mental health disorders and PTSD [57].

The final part of the questionnaire investigated attitudes toward the impact of the pandemic on patients that require chronic care due to NCDs. According to FPs’ opinions, the pandemic has a substantial impact on various aspects of disease management and overall levels of provided healthcare. They believe that patients have limited availability of primary care, as well as access to specialist examinations and diagnostic procedures. Moreover, preventive care and confirmed new diagnosis of NCDs is severely disrupted, according to the majority of the participants. These results are in accordance with a global investigation conducted by WHO in May 2020, in which 75% of the included countries reported significant disruption of NCD services like rehabilitation and palliative care, mostly due to the various changes in healthcare and insufficient staff because of the pandemic [58]. In addition, 59% of countries reported some degree of restriction in outpatient NCD services, also in accordance with our study population’s attitudes. Furthermore, our results support the observation that lockdowns potentially have induced exposure to negative behavioral habits that could have impacted further on negative health outcomes in these patients [58,59].

Since FPs are responsible for a large part of the health care of NCD patients, including early detection through screening programs, these opinions have additional relevance due to their first‑hand insight into primary care experiences. Since the pandemic could have an additional impact on NCD patients’ health due to postponed surgical procedures, questionable medication adherence and halted NCD research, it is crucial to raise awareness about this problem and minimize disruptions and accessibility to health services [58,60].

As this is a cross-sectional study, any causality between the results cannot be assumed. Furthermore, the number of patients in care, area of work (urban or rural/islands) and increased personal risk of COVID-19 due to higher age or presence of relevant NCDs was self‑assessed by the participants, and not retrieved from official records. Therefore, some information could have been misinterpreted, though we assume that physicians can properly estimate these details about their practice and personal health. Since this was an online survey, correct answers to knowledge questions could have been searched for, and therefore provide a false level of knowledge, although anonymity in this survey was guaranteed, and appropriate notes before the knowledge questions were included in order to prevent these circumstances. Finally, personal information regarding experienced COVID-19 infection or self-isolation measures were not investigated due to potential anonymity violation.

## 5. Conclusions

In conclusion, the results of this nationwide study show that the COVID-19 pandemic could have a significant deleterious impact on FPs’ mental health. Public health policies should make an additional emphasis on the extensive workload FPs carry during this crisis, with possible implementation of supportive care programs and work-relief strategies. Furthermore, FPs expressed good overall knowledge about the COVID-19 pandemic, but some important aspects of disease management and patient triage could have been better known about, especially in the current scenario, almost a year since the first cases of COVID-19 were recorded. As there is a strong desire among FPs to attend more COVID-19 related educational seminars, and due to the possible protective role of knowledge on mental health distress levels, implementation of proper training programs could be one of the key responses. Finally, with a probably significant pandemic impact on overall health and continuity of care for NCD patients, convenient strategies and preventive programs to tackle these problems could be introduced.

Hopefully, in time, vaccination programs will resolve this crisis and numerous aspects of life will again be normalized. However, important notes should be taken from this experience for similar future challenges and possible new epidemics. Early implementation of proper training programs for FPs, raised awareness regarding mental health disorders and impact on NCD patients in times of crisis are some important aspects that should be addressed.

## Figures and Tables

**Table 1:** Socio-demographic and COVID-19-related knowledge characteristics of study population.

Parameter	Female Physicians (*N* = 491)	Male Physicians (*N* = 122)	Total Population (*N* = 613)	*p* *
Age (years)	44.0 (35.0–55.0)	43.0 (36.0–55.0)	44.0 (35.0–55.0)	0.279
Work experience (years)	13.0 (6.2–25.7)	14.5 (7.0–27.0)	13.0 (7.0–26.0)	0.630
Increased personal risk of COVID-19	138 (28.1)	34 (27.9)	172 (28.1)	0.958
Work experience in COVID-19 center	326 (66.4)	85 (69.7)	411 (67.0)	0.491
Occupation				
Family physician	415 (84.5)	114 (93.4)	529 (86.3)	0.010
Family medicine resident	76 (15.5)	8 (6.6)	84 (13.7)	
Practice localization				
Urban area	338 (68.8)	78 (63.9)	416 (67.9)	0.299
Rural area/islands	153 (31.2)	44 (36.1)	197 (32.1)	
Main source of COVID-19 information				
Online medical databases	65 (13.2)	20 (16.4)	85 (13.9)	0.647
Colleagues health professionals	73 (14.9)	18 (14.8)	91 (14.8)	
WHO/CIPH recommendations	304 (61.9)	69 (56.6)	373 (60.0)	
News (online, television)	29 (5.9)	7 (5.7)	36 (5.9)	
Other	20 (4.1)	8 (6.6)	28 (4.6)	
Confident in personal COVID-19 knowledge	192 (39.1)	53 (43.4)	245 (40.0)	0.382
Knowledge test score	16.0 (14.2–17.0)	15.0 (14.0–17.0)	16.0 (14.0–17.0)	0.115

Data are presented as median (interquartile range) or *N* (%) where appropriate. COVID-19—coronavirus disease 2019; CIPH—Croatian Institute of Public Health. * chi-square test or Mann-Whitney U test.

**Table 2:** Mental health questionnaire characteristics of study population according to gender.

Parameter	Female Physicians (*N* = 491)	Male Physicians (*N* = 122)	Total Population (*N* = 613)	*p* *
Mental health questionnaires scores				
Perceived Stress Scale	21.0 (16.0–27.0)	16.0 (12.0–19.0)	20.0 (16.0–26.0)	<0.001
Impact on Event Scale-COVID19	26.0 (15.0–39.0)	15.0 (4.0–23.0)	24.0 (14.0–36.2)	<0.001
HADS-Anxiety	10.0 (6.0–13.0)	8.0 (4.0–12.0)	9.0 (5.0–12.0)	0.001
HADS-Depression	8.0 (4.0–10.0)	6.5 (3.0–10.0)	8.0 (4.0–10.0)	0.005
Mental health questionnaires categories				
Perceived Stress Scale				
Low perceived stress	40 (8.1)	35 (28.7)	75 (12.2)	<0.001
Moderate perceived stress	315 (64.2)	75 (61.5)	390 (63.6)	
High perceived stress	136 (27.7)	12 (9.8)	148 (24.1)	
Impact on Event Scale-COVID19				
Subclinical/Mild PTSD symptoms	241 (49.1)	95 (77.9)	336 (54.8)	<0.001
Moderate PTSD symptoms	153 (31.2)	17 (13.9)	170 (27.7)	
Severe PTSD symptoms	97 (19.8)	10 (8.2)	107 (17.5)	
HADS-Anxiety subscale				
Normal result	185 (37.7)	58 (47.5)	243 (39.6)	0.047
Borderline case	94 (19.1)	26 (21.3)	120 (19.6)	
Abnormal	212 (43.2)	38 (31.1)	250 (40.8)	
HADS-Depression subscale				
Normal	223 (45.4)	69 (56.6)	292 (47.6)	0.009
Borderline case	147 (29.9)	38 (31.1)	185 (30.2)	
Abnormal	121 (24.6)	15 (12.3)	136 (22.2)	

Data are presented as median (interquartile range) or *N* (%) where appropriate; COVID-19—Coronavirus disease 2019; PTSD—Post-Traumatic Stress Disorder; HADS—Hospital Anxiety and Depression Scale; * chi-square test or Mann-Whitney U test.

**Table 3:** Correlation of mental health questionnaire scores with other relevant parameters.

Parameter	PSS *r* (*p* *)	HADS-A *r* (*p* *)	HADS-D *r* (*p* *)	IES-COVID19 *r* (*p* *)
Age (years)	0.106 (0.009)	−0.011 (0.776)	0.111 (0.006)	0.195 (<0.001)
Work experience (years)	0.110 (0.006)	0.024 (0.554)	0.128 (0.001)	0.212 (<0.001)
Patients in care (*N*) ^†^	0.128 (0.002)	0.119 (0.003)	0.135 (0.001)	0.050 (0.237)
Knowledge score	−0.064 (0.114)	−0.131 (0.001)	−0.048 (0.239)	0.045 (0.267)
PSS	-	0.411 (<0.001)	0.390 (<0.001)	0.592 (<0.001)
HADS-A	0.411 (<0.001)	-	0.805 (<0.001)	0.418 (<0.001)
HADS-D	0.390 (<0.001)	0.805 (<0.001)	-	0.382 (<0.001)

PSS—Perceived Stress Scale; IES-COVID-19—Impact on Event Scale-coronavirus disease 2019; HADS-A—Hospital Anxiety and Depression Scale-Anxiety subscale; HADS-D—Hospital Anxiety and Depression Scale-Depression subscale. * Spearman rank correlation. ^†^ according to *N* = 567.

**Table 4:** Multiple linear regression analysis of independent predictors for mental health questionnaire scores set as dependent variables.

Variables	Β *	SE ^†^	t-Value	*P*
Model 1—HADS-Anxiety score (F-ratio = 3.11; *p* = 0.005)				
Age (years)	−0.03	0.02	−1.37	0.169
Increased personal risk of COVID-19	1.02	0.52	1.96	0.050
Practice localization (urban vs. rural/island area)	−0.001	0.42	−0.001	0.998
Work experience in COVID-19 center	0.06	0.42	0.14	0.885
Gender (male vs. female)	1.51	0.49	3.01	0.003
Knowledge score	−0.31	0.11	−2.76	0.006
Model 2—HADS-Depression score (F-ratio = 5.18; *p* < 0.001)				
Age (years)	0.03	0.02	1.43	0.152
Increased personal risk of COVID-19	0.92	0.45	2.02	0.044
Practice localization (urban vs. rural/island area)	0.41	0.37	1.09	0.275
Work experience in COVID-19 center	1.24	0.37	3.36	<0.001
Gender (male vs. female)	1.16	0.43	2.67	0.007
Knowledge score	−0.07	0.09	−0.78	0.435
Model 3—IES-COVID19 score (F-ratio = 22.2; *p* < 0.001)				
Age (years)	0.11	0.06	1.88	0.060
Increased personal risk of COVID-19	9.39	1.55	6.06	<0.001
Practice localization (urban vs. rural/island area)	2.21	1.26	1.74	0.081
Work experience in COVID-19 center	1.22	1.25	0.97	0.329
Gender (male vs. female)	11.6	1.47	7.84	<0.001
Knowledge score	−0.37	0.32	−1.15	0.250
Model 4—Perceived Stress Scale score (F-ratio = 23.2; *p* < 0.001)				
Age (years)	−0.001	0.03	−0.28	0.775
Increased personal risk of COVID-19	4.05	0.69	5.82	<0.001
Practice localization (urban vs. rural/island area)	−0.09	0.56	−0.15	0.897
Work experience in COVID-19 center	1.77	0.57	3.13	0.002
Gender (male vs. female)	6.21	0.66	9.34	<0.001
Knowledge score	−0.33	0.15	−2.27	0.023

HADS—Hospital Anxiety and Depression Scale; IES-COVID-19—Impact on Event Scale-coronavirus disease 2019. * unstandardized coefficient β. ^†^ standard error.

## Data Availability

The data presented in this study are available on request from the corresponding author. The data are not publicly available due to ethical restrictions.

## Supplementary Materials

The following are available online at https://www.mdpi.com/1660-4601/18/4/2093/s1, Table S1: Correct answers on knowledge questionnaire questions in total study population, Table S2: Attitudes regarding COVID-19 of the total study population (*N* = 613), Table S3: Practices regarding COVID-19 of the total study population (*N* = 613), Table S4: Attitudes regarding impact of the pandemic on patients that require chronic care due to noncommunicable diseases (*N* = 613).
