# Supplementary material for: Family Physicians’ Standpoint and Mental Health Assessment in the Light of COVID-19 Pandemic—A Nationwide Survey Study
Source: Int J Environ Res Public Health. 2021 Feb 21;18(4):2093. doi: 10.3390/ijerph18042093 (PMC7924834; doi:10.3390/ijerph18042093)
Supplement: Supplementary file 1 [file ijerph-18-02093-s001.pdf]

Supplementary material

Table S1. Correct answers on knowledge questionnaire questions in total study population.

Knowledge question	Correct answer rate (N=613)
**K1.** The main clinical symptoms of COVID-19 are fever, fatigue and cough.	602 (98.2)
**K2.** Complications of COVID-19 include increased formation of blood clots and acute kidney injury.	544 (88.7)
**K3.** Unlike common cold, stuffy nose, runny nose, and sneezing are less common in COVID-19 patients.	419 (68.4)
**K4.** There is currently no effective cure for COVID-19, but early symptomatic treatment can help most patients recover from the infection.	552 (90.0)
**K5.** COVID-19 mainly spreads via respiratory droplets of infected individuals through coughing and sneezing.	606 (98.9)
**K6.** The virus can be transmitted with fecal-oral pathway.	212 (34.6)
**K7.** Patients with COVID-19 cannot transmit the virus to others if the fever is not present.	577 (94.1)
**K8.** COVID-19 is transmitted indirectly by touching surfaces and devices contaminated with the secretions of the patient and then by touching the mouth, nose, or eye.	591 (96.4)
**K9.** Basic Reproductive Rate (R0) for COVID-19 is estimated to be between 2 and 4.	296 (48.3)
**K10.** Patients may still be infectious after they improve clinically.	544 (88.7)
**K11.** Vomiting and diarrhea are possible, but not common symptoms of COVID-19.	588 (95.9)
**K12.** There is moderate risk in transmitting COVID-19 in dealing with domestic animals.	244 (39.8)
**K13.** The incubation period of the disease is from 1 to 14 days.	579 (94.5)
**K14.** Antibiotics and usual antiviral drugs are first treatment of choice for COVID-19.	385 (62.8)
**K15.** For COVID-19, around 10-15% cases progress to severe disease that requires oxygen treatment, while around 5% become critically ill, requiring ventilation.	547 (89.2)
**K16.** In close contacts of a confirmed COVID-19 patient who has no signs of disease, testing for the new coronavirus is not routinely performed either at the beginning or during the isolation.	592 (96.6)
**K17.** Mortality from COVID-19 appears to be lower than from seasonal influenza.	362 (59.1)
**K18.** As it proposes low risk for severe disease, it is not necessary for children and young adults to take measures in preventing COVID-19 infection.	574 (93.6)
**K19.** Pregnancy facilitates the severity of COVID-19 and pregnant women are more likely to need intensive care if severely ill.	97 (15.8)
**K20.** Fatigue, cough and loss of taste and/or smell are some of the symptoms that can persist even after the acute phase of the disease.	608 (99.2)

COVID-19- coronavirus disease 2019.
Data are presented as N (%).

Table S2. Attitudes regarding COVID-19 of the total study population (N=613).

Attitudes	Fully disagree	Disagree	Neither agree nor disagree	Agree	Fully agree
**A1.** Face mask can prevent viral transmission.	7 (1.1)	21 (3.4)	41 (6.7)	296 (48.3)	248 (40.5)
**A2.** PPE is not enough to protect us from infection.	62 (10.1)	293 (47.8)	76 (12.4)	157 (25.6)	25 (4.1)
**A3.** I am afraid of getting the disease during work.	42 (6.9)	82 (13.4)	118 (19.2)	282 (46.0)	89 (14.5)
**A4.** I am concerned about the social isolation present during the COVID-19 pandemic.	24 (3.9)	53 (8.6)	67 (10.9)	330 (53.8)	139 (22.7)
**A5.** I am concerned about the risk that COVID-19 poses to my family and friends.	17 (2.8)	33 (5.4)	56 (9.1)	337 (55.0)	170 (27.7)
**A6.** When a vaccine for the COVID-19 is developed, the spread of the epidemic can be significantly reduced.	20 (3.3)	43 (7.0)	75 (12.2)	305 (49.8)	170 (27.7)
**A7.** If the COVID-19 vaccine becomes available in the next 6 months, I would vaccinate myself.	54 (8.8)	72 (11.7)	172 (28.1)	189 (30.8)	126 (20.6)
**A8.** PPE is not available in sufficient amount for the family physicians.	29 (4.7)	92 (15.0)	60 (9.8)	264 (43.1)	168 (27.4)
**A9.** Family physicians are well trained and educated regarding usage of PPE.	46 (7.5)	159 (26.0)	113 (18.4)	211 (34.4)	84 (13.7)
**A10.** Hydroxychloroquine treatment is an excellent measure for COVID-19 prevention.	252 (41.1)	185 (30.2)	146 (23.8)	24 (3.9)	6 (1.0)
**A11.** Family physicians are prescribing more antibiotics in comparison with the time before pandemic.	112 (18.3)	145 (23.6)	88 (14.4)	179 (29.2)	89 (14.5)
**A12.** This year, I am planning to get a seasonal influenza vaccination.	68 (11.1)	66 (10.8)	49 (8.0)	199 (32.4)	231 (37.7)
**A13.** I regularly follow updates with newest information on COVID-19.	16 (2.6)	28 (4.6)	58 (9.5)	350 (57.1)	161 (26.3)
**A14.** I have read articles published in scientific journals related to COVID-19.	27 (4.4)	104 (17.0)	76 (12.4)	276 (45.0)	130 (21.2)
**A15.** I would like to attend more educational seminars on COVID-19 if they were organized in my area or through online applications.	28 (4.6)	32 (5.2)	95 (15.5)	322 (52.5)	136 (22.2)

COVID-19- coronavirus disease 2019; **PPE-** personal protective equipment
Data are presented as N (%)

Table S3. Practices regarding COVID-19 of the total study population (N=613).

Practices	Never	Sometimes	Often	Always
**P1.** I clean my hands with soap and water for at least 30 s regularly and thoroughly.	4 (0.7)	25 (4.1)	254 (41.4)	330 (53.8)
**P2.** I cover my mouth and nose with a tissue and/or bend my elbow when I cough or sneeze.	4 (0.7)	17 (2.8)	174 (28.3)	418 (68.2)
**P3.** I wash my nose with a saline solution.	223 (36.4)	194 (31.6)	147 (24.0)	49 (8.0)

**P4.** I wash my hands before wearing and after removing the mask.	20 (3.3)	161 (26.3)	232 (37.8)	200 (32.6)
**P5.** When I am leaving home, I wear a mask and change it regularly.	12 (2.0)	59 (9.6)	233 (38.0)	309 (50.4)
**P6.** When I am leaving home, I wear protective gloves and change them regularly.	249 (40.6)	216 (35.2)	104 (17.0)	44 (7.2)
**P7.** I am avoiding social gatherings.	8 (1.3)	67 (10.9)	231 (37.7)	307 (50.1)
**P8.** I canceled or postponed meetings with friends, and going to social and sporting events.	7 (1.1)	91 (14.9)	244 (39.8)	271 (44.2)
**P9.** I educated my family and friends about COVID-19 prevention measures.	3 (0.5)	26 (4.2)	271 (44.2)	313 (51.1)
**P10.** I regularly clean and disinfect surfaces at my workplace.	7 (1.1)	34 (5.5)	259 (42.3)	313 (51.1)
**P11.** I wear a mask while interacting with patients.	0 (0.0)	4 (0.7)	99 (16.2)	510 (83.2)
**P12.** I wear protective gloves when interacting with patients.	30 (4.9)	127 (20.7)	242 (39.5)	214 (34.9)
**P13.** I perform hand hygiene before and after touching the patients for examination.	1 (0.2)	10 (1.6)	128 (20.9)	474 (77.3)
**P14.** I practice social distancing, and keeping at least 1 m away from patients and other health care professionals.	2 (0.3)	27 (4.4)	188 (30.7)	396 (64.6)
**P15.** I educate my patients about preventative measures for COVID-19.	2 (0.3)	19 (3.1)	198 (32.3)	394 (64.3)

COVID-19- coronavirus disease 2019
Data are presented as N (%)

Table S4. Attitudes regarding impact of the pandemic on patients that require chronic care due to noncommunicable diseases (N=613).

**Attitudes**	**Fully disagree**	**Disagre e**	**Neither agree nor disagree**	**Agree**	**Fully agree**
**A_NCD1.** During the COVID-19 pandemic, NCD patients had limited accessibility to family physicians.	122 (19.9)	130 (21.2)	54 (8.8)	247 (40.3)	60 (9.8)
**A_NCD2.** During the average day, family physician service is provided to lesser number of NCD patients.	195 (31.8)	229 (37.4)	39 (6.4)	103 (16.8)	47 (7.6)
**A_NCD3.** I have more time to devote to the NCD patient.	257 (41.9)	200 (32.6)	90 (14.7)	47 (7.7)	19 (3.1)
**A_NCD4.** There is decreased number of newly diagnosed NCD patients in comparison with time before the pandemic.	30 (4.9)	61 (10.0)	81 (13.2)	297 (48.5)	144 (23.5)
**A_NCD5.** COVID-19 pandemic aggravates the availability of necessary specialist conciliatory examinations.	15 (2.4)	44 (7.2)	59 (9.6)	295 (48.1)	200 (32.6)
**A_NCD6.** COVID-19 pandemic aggravates the availability of necessary diagnostic procedures.	11 (1.8)	52 (8.5)	56 (9.1)	292 (47.6)	202 (33.0)

**A_NCD7.** COVID-19 pandemic aggravates the availability of chronic therapy.	217 (35.4)	285 (46.5)	51 (8.3)	31 (5.1)	29 (4.7)
**A_NCD8.** During the COVID-19 pandemic, NCD patients experienced illness exacerbation.	62 (10.1)	162 (26.4)	230 (37.5)	117 (19.1)	42 (6.9)
**A_NCD9.** NCD patients died in larger numbers from their illnesses in comparison with time before the pandemic.	74 (12.1)	141 (23.0)	253 (41.3)	105 (17.1)	40 (6.5)
**A_NCD10.** NCD patients take their therapy less regularly and properly.	123 (20.0)	180 (29.4)	115 (18.8)	150 (24.5)	45 (7.3)
**A_NCD11.** NCD patients are in fear of COVID-19 infection.	7 (1.1)	11 (1.8)	43 (7.0)	328 (53.5)	224 (36.5)
**A_NCD12.** During the pandemic, harmful, unhealthy habits of NCD patients, such as substance addiction and unhealthy eating habits, worsened.	19 (3.1)	62 (10.1)	190 (31.0)	273 (44.5)	69 (11.3)
**A_NCD13.** NCD patients adhere properly to preventive epidemiological measures.	27 (4.4)	183 (29.8)	214 (34.9)	168 (27.4)	21 (3.4)
**A_NCD14.** Due to the COVID-19 pandemic, NCD patients will have to spend more money on their disease.	48 (7.8)	126 (20.6)	182 (29.7)	206 (33.6)	51 (8.3)
**A_NCD15.** NCD patients will suffer from a lack of physical activity, caused by limited opportunities during pandemic.	19 (3.1)	60 (9.8)	171 (27.9)	232 (37.8)	131 (21.4)

NCD- noncommunicable disease; COVID-19- coronavirus disease 2019
Data are presented as N (%)
